# Trace Elements in Parenteral Nutrition: Considerations for the Prescribing Clinician

**DOI:** 10.3390/nu9050440

**Published:** 2017-04-28

**Authors:** Jennifer Jin, Leanne Mulesa, Mariana Carrilero Rouillet

**Affiliations:** 1 Division of Gastroenterology, Department of Medicine, Royal Alexandra Hospital, University of Alberta, Edmonton, AB T5H 3V9, Canada; 2 Alberta Health Services, Edmonton, AB T6G 2B7, Canada; leanne.mulesa@albertahealthservices.ca; 3 Division of Gastroenterology, Department of Medicine, University of Alberta, Edmonton, AB T6G 2B7, Canada; marianacarrilero@gmail.com

**Keywords:** parenteral nutrition, trace elements, copper, chromium, zinc, selenium, manganese

## Abstract

Trace elements (TEs) are an essential component of parenteral nutrition (PN). Over the last few decades, there has been increased experience with PN, and with this knowledge more information about the management of trace elements has become available. There is increasing awareness of the effects of deficiencies and toxicities of certain trace elements. Despite this heightened awareness, much is still unknown in terms of trace element monitoring, the accuracy of different assays, and current TE contamination of solutions. The supplementation of TEs is a complex and important part of the PN prescription. Understanding the role of different disease states and the need for reduced or increased doses is essential. Given the heterogeneity of the PN patients, supplementation should be individualized.

## 1. Introduction

Trace elements (TE) are essential components of complexes required for fundamental processes such as enzymatic reactions. There are TEs that are well established as essential in human physiology, and other TEs whose roles and requirements have yet to be defined. In 1979, the Nutrition Advisory Group of the American Medical Association (NAG-AMA) published guidelines submitted to the Food and Drug Administration (FDA) for daily TEs considered essential for human health in parenteral nutrition: zinc, copper, manganese, and chromium [1]. A few years later, selenium was added to a subsequent review [2]. As patients were maintained on home parenteral nutrition (HPN) for longer durations of time, additional light was shed on TEs. In the following decades, as more information came from toxicity and balance studies, as well as reports in the literature of PN contamination, further adjustments to recommended doses were made [3]. In critical illness, the processes of inflammation, infection, and oxidative stress increase the metabolic requirements for certain trace elements. Conversely, in conditions of organ dysfunction, certain reductions in trace elements are recommended [4]. Despite expert agreement that copper, manganese, and chromium doses be reduced, the FDA has not updated the 1979 guidelines [2,5]. There are currently a variety of pre-mixed multi-TE combinations that are commercially available for addition to parenteral nutrition (PN) solutions. Many of these still contain doses of TEs that are not in keeping with the current recommendations from expert groups [5,6]. The following is a review of TEs that are currently supplemented in PN, and a brief review on TEs that are not routinely added in North America but whose importance should not be overlooked. The aim of this paper aims to summarize the most recent recommendations to help guide the clinician prescribing PN in acute care and long-term settings.

### Methods

A literature search was conducted using PubMed until February 2017 and additional citations were hand-searched. Search terms included “trace elements in parenteral nutrition”, and “parenteral nutrition” plus: “-copper”, “-chromium”, “-zinc”, “-selenium”, and “-manganese”. Only articles in English were included. Articles addressing trace elements in both the acute care and long-term PN setting were included. The most up to date guidelines and recommendations from national nutrition and critical care societies were also reviewed.

## 2. Trace Elements

### 2.1. Copper

Copper is essential as an enzymatic cofactor in processes involving connective tissue formation, iron metabolism and hematopoesis, and central nervous system function [4]. More than 90% of copper in the blood is bound to ceruloplasmin and the rest is bound to albumin and amino acids [7]. Eighty percent of copper is excreted in the bile and the remainder is excreted in the urine [8]. In the context of PN, copper may have increased urinary excretion, as copper is complexed to amino acids [9].

In copper deficiency, serum copper and ceruloplasmin levels are low but if the deficiency is not severe, these levels can be normal and thus do not reflect copper status in the body. Additionally, as ceruloplasmin is an acute phase reactant, ceruloplasmin and copper levels can be elevated in inflammatory states even in the setting of marginal copper deficiency [7]. In a recent study of 166 patients, copper levels had no correlation with C-reactive protein (CRP) levels, unlike zinc, selenium, and albumin, which were negatively correlated with CRP [10]. This highlights the difficulty in interpreting serum copper levels in the setting of inflammation.

Clinical manifestations of copper deficiency are pancytopenia (including hypochromic anemia unresponsive to iron supplementation), skeletal abnormalities, myocardial disease, depigmentation of hair, and neurologic abnormalities [11]. Furhman et al. summarized 14 case reports of PN-related copper deficiency and in addition to neutropenia and/or anemia, 3 of the 14 patients also had thrombocytopenia [12]. Anemia and neutropenia were the first signs of copper deficiency after an average of 11 months. In one patient, pancytopenia developed 8 weeks after the removal of copper from PN [13]. Thrombocytopenia in the reported cases improved as early as 2–6 weeks after supplementation [14].

Acute copper toxicity results in vomiting, diarrhea, acute kidney injury, hepatic necrosis, and death [3]. Wilson’s disease, a genetic abnormality of cellular transport, is an example of chronic copper toxicity. Symptoms of chronic toxicity include neurological disorders, renal insufficiency, and cirrhosis [3]. Blaszyk et al. performed biopsies on 28 patients on long-term PN with intestinal failure-associated liver disease as well as 10 controls with drug-induced cholestatic liver disease and showed that 89% of PN patients versus all controls had mildly elevated hepatic tissue copper, but 29% of the PN patients had levels above 250 mcg/g, the diagnostic threshold for Wilson’s disease [15]. The amount of hepatic copper did not correlate with the duration of PN or serum copper levels, but patients with significant cholestasis accumulated more copper than patients with no or minimal cholestasis. Copper accumulation can contribute to and cause liver damage [3].

Copper is found as a contaminant in components of PN such as amino acids and sterile water [16]. Current multiple TE solutions available in North America contain approximately 1 mg/day of copper. In a study of long-term PN patients, copper at 1 mg/day resulted in 22.5% of patients having a level above the normal range [17]. The most recent American Society of Parenteral and Enteral Nutrition (A.S.P.E.N.) recommendations call for the daily dose of copper to be lowered to 0.3–0.5 mg/day [3]. In a balance study in PN patients, 0.15 mg/day appeared to be an optimal dose for patients with cholestasis and in patients with diarrhea, 0.4–0.5 mg/day appeared to maintain balance [18]. Increased copper losses can be seen during nasogastric suction [19], and burn patients may require additional copper [20].

### 2.2. Chromium

Trivalent chromium is an essential trace element that is a component of metalloenzymes and functions as a coenzyme in various metabolic reactions [21,22]. Chromium has a role in the regulation of glucose levels and insulin resistance [4]. It is transported in the blood bound to transferrin and albumin while also competing with iron for a binding site on transferrin [21]. Chromium promotes the actions of insulin, enhances its activity in peripheral tissue, and reduces insulin requirements [21].

Signs and symptoms of chromium deficiency include glucose intolerance refractory to insulin, hyperlipidemia, elevated plasma free fatty acids, weight loss, peripheral neuropathy, and encephalopathy [21,23]. Patients receiving PN not supplemented with chromium have been found to have chromium deficiency after glucose intolerance developed [21]. There may be increased chromium needs during pregnancy [21]. Chromium deficiency has been described in as short a period as five months in unsupplemented PN [8].

Although the evidence of toxicity of trivalent chromium is limited, the Food and Nutrition Board of the Institute of Medicine has acknowledged that high intakes of supplemental chromium has the potential for adverse effects and advised caution in supplementation. Chromium given intravenously is excreted by the kidneys, so attention should be taken in supplementation in renal insufficiency [8,21]. In adults, there are no reported cases of patients on long-term PN developing chromium toxicity. A pediatric study on PN patients showed that glomerular filtration rate was inversely related to serum chromium concentration; this was partially reversed with chromium discontinuation although direct causal effect could not be made [24].

Excessive serum concentration of chromium has been associated with the long-term use of TE products. Btaiche found that 96% of serum chromium measurements in a study of HPN patients were above reference range, with the mean daily dose being 9.33 ± 0.42 mcg [17]. Elevated tissue levels have been found in doses of 14 mcg/day [25]. The current A.S.P.E.N. recommendation is that 10–15 mcg/day of chromium is supplemented in PN [3]. During shortages, chromium does not need to be supplemented unless clinical signs and symptoms of deficiency appear [24]. Some components of PN solutions have been found to be contaminated with chromium and these contaminants can increase the amount delivered by 10 to 100% [3,21]. Pluhator-Murton et al. [16] calculated that, in a 2 litre PN solution, 15 mcg of chromium is present as a contaminate. For this reason, the serum monitoring of chromium is necessary in patients who receive long-term PN because of the risk of toxicity. While there is suggestion that lower doses are sufficient and that even the amount in PN from contamination alone may be adequate [17], the rationale for recommending supplementation remains because current levels of PN contamination are unclear, as are the consequences of chromium toxicity.

Reliable tests to measure chromium are limited. Blood chromium does not readily equilibrate with tissue chromium stores [21]; plasma and serum levels may not be good indicators of chromium status. Moreover, in patients receiving PN, plasma or serum levels may reflect the amount of infused chromium but not the tissue status. As well, there are issues with laboratory and handling procedures contributing to erroneous results [21]. Although high urinary levels may be a good indicator of exposure to excessive amounts of chromium, they do not provide a useful predictor of tissue status [21,26]. Chromium levels may be reduced in acute illness [21]. In patients with abnormal glucose tolerance, chromium should be measured. If chromium deficiency is detected and blood glucose control improves with supplementation, this would support the diagnosis of deficiency.

### 2.3. Zinc

Zinc has a ubiquitous subcellular presence and is involved in catalytic, structural, and regulatory roles in the human body [4]. It has been identified as a part of about 120 enzymes [27]. Because it is involved in so many processes, clinical features of zinc deficiency are non-specific and include the development of eye and skin lesions, growth retardation, alopecia, and diarrhea [4]. While 86% of total body zinc is stored in skeletal muscle and bone, only 0.1% is found in plasma [4], which is kept at a tightly regulated concentration of 12–18 mcmol/L.

Diagnosis of zinc deficiency is difficult. During times of stress and infection, plasma zinc levels are decreased [4]. One hypothesis is that zinc is taken up into vital organs during these times to support metabolic functions [28,29]. Besecker et al. [30] observed that plasma zinc levels declined more in a cohort of patients with sepsis versus a critically ill control group without infection, despite similar severity-of-illness scores. Levels can be low in hypoalbuminemia as it is bound to albumin and alpha-macroglobulins in the blood [31]. Zinc has been found to correlate negatively with CRP [10]. A review found that, in minor illness (CRP < 15 mg/L), zinc was decreased by 10%, whereas major illness (CRP 100–200 mg/L) caused a 40%–60% decrease [32]. Zinc can decrease by 50% within 6 h post-operatively [33]. Acute zinc toxicity can occur with doses of more than 200 mg orally, which leads to abdominal pain, vomiting, and diarrhea. In a case report where a patient inadvertently received 7.4 g of zinc in her PN over 60 h, she developed hypotension, pulmonary edema, jaundice, and oliguria [34]. In chronic zinc toxicity from oral zinc, serum copper levels can decrease due to interference with oral absorption, neutropenia can develop and immune function may be affected [3].

The current A.S.P.E.N. recommended supplementation in PN for zinc is 2.5–5 mg/day [3]. Patients with excessive gastrointestinal (GI) losses, sepsis, hypercatabolic states, and burns require additional supplementation [35,36]. Amino acid infusions also increase urinary zinc loss [27]. Wolman et al. [37] showed that, in patients with no oral intake on PN alone, about 3 mg/day of zinc maintained zinc balance in the absence of GI losses. Patients with enterocutaneous fistulae, diarrhea, and ostomy losses may require 12–17 mg per L of fluid lost [3,37]. In patients with burns, up to 36 mg/day of supplementation has been shown to provide benefit including a reduction in infectious complications, without toxicity [27]. In their review of 26 HPN patients, Btaiche et al. [17] found that short bowel syndrome (SBS) patients required a higher zinc dose of about 9 mg/day vs. 6.7 mg/day for non-SBS patients. These doses maintained normal serum zinc concentrations in about 90% of patients.

In cases of zinc deficiency, clinical improvement is quite rapid, with results seen in 2 days and resolution in 2 weeks [38].

### 2.4. Selenium

Selenium is a trace element whose key roles in human health include antioxidant, anti-inflammatory, and immunological activities. Selenium functions via proteins known as selenoproteins [39,40]. The most important selenoprotein is glutathione peroxidase (GSH-Px), which assists the body in defending against oxidative stress by reducing cell membrane damage from lipid hydroperoxides and hydrogen peroxide. Selenium may also have a role in anti-inflammatory activity by inhibiting nuclear transcription factor kappaB [41]. Selenium also plays a role in the regulation of thyroid hormone metabolism via three selenium dependent enzymes [42]. It is through this wide range of biochemical and physiologic functions that selenium has become an essential nutrient in all forms of nutrition, including parenteral nutrition.

Selenium deficiency was first recognized as Keshan disease, a cardiomyopathy seen in those with selenium deficient diets in various parts of the world [43,44]. Over time, selenium deficiency has also been described in patients receiving parenteral nutrition lacking in selenium. This includes reports of cardiomyopathy, skeletal muscle myopathy, macrocytic anemia, and abnormalities in hair and nails [43]. Other causes of selenium deficiency, however, are possible. Plasma levels of selenium have been shown to be decreased during acute illnesses such as thermal injury, trauma, post surgery, and systemic inflammatory response syndrome [43]. This increased demand for selenium during an acute phase response can potentially contribute to a selenium deficiency, especially in an already nutritionally compromised individual. Additionally, the critically ill are also subject to various therapies and medications that increase the risk of developing a selenium deficiency. Some examples include continuous renal replacement therapy (CRRT), high urine output, diarrhea/fistula output, multiple drains, and medications such as corticosteroids and statins [45]. In a recent study of HPN patients, Uzzan et al. [46] found that 21% of 73 patients in the program had a low serum selenium and that low serum selenium was independently associated with a higher risk of developing a serious infection.

Although not optimal, monitoring of selenium status in individuals is primarily accomplished with plasma or serum selenium levels, as it is most readily available. Levels should be accompanied by a C-reactive protein, as there are obligatory decreases in serum selenium during the acute phase of illness. Other more accurate measures of selenium status exist, such as plasma (GSH-Px), erythrocyte GSH-Px, and hair and nail selenium levels, but these are often not readily available or are used only in research settings [43]. For long-term PN patients, a regular assessment for physical signs of selenium deficiency should be accompanied by a plasma selenium level if a deficiency is suspected.

Appropriate dosing is dependent on age, baseline nutritional history, and overall clinical status. Past recommendations for dosages have fluctuated, but the most recent recommendation from A.S.P.E.N. is 60–100 mcg/day for PN trace element formulations [3,39,43]. Btaiche et al. [17] found in their review of HPN patients that a dose of 70 mcg/day, which was above the A.S.P.E.N. guidelines at the time, resulted in about 38% of serum selenium to be below normal in both SBS and non-SBS patients. It is strongly recommended that selenium be routinely added to all PN solutions. Some evidence suggests doses up to 400 mcg/day for burn patients on parenteral therapy may be beneficial. A systematic review found that parenteral selenium supplementation in sepsis in comparison to a placebo was associated with lower mortality [47]. In another systematic review by Huang et al. [48], 965 patients in total were included and the study found that parenteral selenium reduced all-cause mortality in critically ill patients with sepsis. However, for critically ill patients in general, the Canadian Clinical Practice Guidelines at present have advised against high dose selenium supplementation, as the evidence is overall inconclusive [49,50]. The risk of selenium toxicity in all patient populations appears to be rare with a tolerable upper limit for oral intake set at 400 mcg/day in the healthy population [42,43]. Signs of toxicity include hair and nail brittleness, peripheral neuropathy, skin rash, fatigue, and GI symptoms [43,51].

Areas for further research with parenteral selenium involve its role in reducing the risk of various chronic diseases such as certain cancers, heart disease, and cognitive decline [42].

### 2.5. Manganese

Manganese is required to activate certain enzymatic reactions and is a component of several metalloenzymes [4]. Manganese is involved in a variety of reactions in processes involving immune function, bone and connective tissue development, reproductive function, neuronal health, and detoxification of free radicals [52]. As 90% is excreted in the bile [53], high levels can develop in PN patients with cholestasis, but elevated levels can be seen in patients with normal liver function as well [43,54,55]. In addition, it is possible that manganese can also contribute to cholestasis [56,57,58,59]. Apart from experimental cases of manganese deprivation, there has only been one reported case of manganese deficiency, which occurred in a child with a very short bowel on PN [60]. As such, the question of whether or not manganese deficiency is clinically relevant has been raised [53].

Conversely, manganese toxicity in PN has been well reported. Excess manganese can lead to deposition in the basal ganglia of the brain, which can been demonstrated on magnetic resonance imaging (MRI) and can manifest symptomatically with Parkinson-like signs and symptoms in addition to other neuropsychiatric symptoms [61,62]. This accumulation can appear as increased symmetric signal intensity on T-1 weighted magnetic resonance images and can regress when manganese supplementation is withdrawn in PN [56,63,64,65,66]. Abdalian et al. [66] studied 16 patients on long-term HPN receiving a multi-TE preparation with a mean manganese dose of 400 μg/day. In 57% of patients, blood manganese levels were above the upper level of normal, and 81% of patients had deposits in their basal ganglia. In a recent study of 68 long-term HPN patients, 97% had manganese levels above the reference range, with a median daily dose of 236 mcg [67].

Takagi et al. [68] conducted a dose finding study with manganese supplementation of 0, 55, 110, and 1100 mcg/day using an on/off study design and measured whole blood and plasma manganese and MRI. Elevated levels above the normal range were found in patients on the higher doses of manganese, but all patients with 55 mcg/day maintained whole blood manganese within the normal range. MRI intensity of the globus pallidus in patients increased in a manganese dose-dependent fashion. The authors concluded that 55 mcg/day was an optimal dose in stable HPN patients.

The doses of manganese found in commercially available multi-TE preparations contain 100–800 mcg of manganese [3,66]. Because of the increasing prevalence of reports of hypermanganesemia in HPN patients, in 2012 A.S.P.E.N. updated its recommended dose from 60–100 mcg/day to a lower dose of 55 mcg/day [3]. This dose of 55 mcg/day has been increasingly recommended by experts [69,70]. An inadvertent source of manganese is from contamination of PN solutions, which can contain up to 38 mcg of manganese per 2 L of PN solution, especially from calcium gluconate, magnesium sulfate, potassium chloride, amino acid, and glucose solutions [16].

Measurement of whole blood manganese is more accurate than plasma or serum levels [71]. However, MRIs can be abnormal even with normal levels of manganese [66], so consideration should be taken to perform MRI in HPN patients, although resources to do so may be limited. Fitzgerald et al. [55] studied inpatients on PN and found, out of the 30 patients on less than 30 days of PN, 2 had elevated levels at Days 14 and 18 of PN. They suggested that the monitoring of levels should be routine for patients receiving PN for more than 30 days.

Due to a lack of a clinically defined manganese deficiency syndrome and the large body of evidence that manganese toxicity is a concern, Howard et al. suggested that Mn supplementation be omitted if clinical signs of toxicity are detected, or when liver enzymes rise to twice the upper limit of normal [25]. There is some support from experts that routine manganese supplementation can be excluded even in the absence of liver disease, particularly if there is ongoing oral intake [53]. However, more robust evidence is required before a general recommendation can be made.

### 2.6. Other Trace Elements

Iodine is needed for thyroid function but it is not routinely supplemented in the US [5]. It is included in many multi-TE products in Europe, Australia, and Canada [6,69,70]. There may be contamination of up to 15–25 mcg/day iodine in PN [71] but that amount is significantly less than the recommended dietary intake of 150 mcg/day orally [3]. In the past, iodine was used topically to clean central venous catheter sites, so some absorption was accounted for by that route [3,8]. However, as practice has moved increasingly to cleaning with chlorhexidine, more research is needed to identify the burden of risk of iodine deficiency [3].

Iron deficiency is common in HPN patients [72], but iron is not routinely available in North America in PN formulations for several reasons, including concerns with stability in fat emulsion [5]. It is available for the addition in PN in other markets. A dose of 1 mg/day has been suggested to maintain levels [69, 73], although ongoing monitoring is required to avoid iron overload [74].

Molybdenum and fluoride are not usually supplemented by multi-TEs in North America. A.S.P.E.N. currently does not recommend their routine addition in PN [3].

Choline is synthesized from methionine and is essential for DNA repair and is a fundamental component of cell membranes [3]. Deficiency may contribute to intestinal failure-associated liver disease and even increase risk for hepatocellular carcinoma [3]. Choline may also reduce catheter thrombosis [2]. It is a required nutrient in the diet but optimal doses need to be clarified. A.S.P.E.N. has supported the routine addition of 550 mg/day to PN, but there is currently no commercially available product available [3, 75].

Carnitine is synthesized from lysine and methionine and is required for the breakdown of lipids to transport fatty acids into the mitochondria [3]. It is not essential in the adult although it may be conditionally essential in the neonate [2, 76]. A.S.P.E.N. recommends the routine addition of 2.5–5 mg/kg/day in PN for neonates [3], but there is no current recommendation for routine supplementation in children and adults.

## 3. Conclusions

Despite the convenience that multi-TE admixtures provide, many products are based on outdated recommendations and provide inadequate or supratherapeutic amounts of TEs. In addition, the PN patient population is heterogeneous and fixed doses may not fit individual requirements. Patients may require increased zinc and selenium if there are increased gastrointestinal losses, copper and manganese should be reduced in patients with cholestasis, and manganese and chromium are frequently found in elevated levels in long-term PN. Table 1 provides a summary of the TE in PN and special considerations.

Recently, new multi-TE products have been made available in Europe and provide the A.S.P.E.N. recommended dose changes to manganese, copper, and chromium. Like many other multi-TEs available in Europe, they also contain iron, molybdenum, iodide, and fluoride, which are not in the standard multi-TE available for addition in North America.

Further research is required to provide guidance on the frequency of monitoring TE in long-term PN and to clarify the relationship of TE levels to actual tissue concentrations. Contamination studies need to be updated. Experts have also called for the FDA to require manufacturers to identify contamination of TE in product labeling, and to set limits on the levels of contamination that are acceptable. Each patient on PN should continue to have their TE levels regularly monitored and have individualized doses determined according to those levels.

## Figures and Tables

**Table 1:** Summary of trace elements (TEs) routinely supplemented in parenteral nutrition (PN) in North America.

Trace Element	Signs and Symptoms of Deficiency	Signs and Symptoms of Toxicity	TE Monitoring	Conditions to Consider Dose Reduction	Conditions Requiring Increased Amounts
Copper	Pancytopenia, skeletal abnormalities, myocardial disease, depigmentation of hair, neurologic abnormalities	Acute: vomiting, diarrhea, acute kidney injury, hepatic necrosis, death Chronic: neurological disorders, renal insufficiency, and cirrhosis	Ceruloplasmin, serum copper Levels be elevated in inflammation	Cholestasis	High gastrointestinal (GI) losses (diarrhea, ostomy outputs, nasogastric suctioning), burn patients
Chromium	Glucose intolerance which may be refractory to insulin, hyperlipidemia, elevated plasma free fatty acids, weight loss, peripheral neuropathy	Not well documented	Plasma or serum chromium, although not a good indicator of tissue levels Levels may be reduced during acute illness	Caution in renal insufficiency	Pregnant patients may have increased needs
Zinc	Eye and skin lesions, growth retardation, alopecia, and diarrhea	Acute: large oral doses can cause abdominal pain, vomiting, and diarrhea Chronic: high oral doses can cause low copper by interference with absorption	Serum zinc Levels may be reduced during inflammation, especially sepsis	N/A	Patients with high GI losses, sepsis, hypercatabolic states, and burns
Selenium	Cardiomyopathy, skeletal muscle myopathy, macrocytic anemia, and abnormalities in hair and nails	Hair and nail brittleness, peripheral neuropathy, skin rash, fatigue, and GI symptoms	Plasma/serum selenium Levels may be reduced during inflammation	N/A	Critical illness, burns, continuous renal replacement therapy, high urine output, diarrhea/fistula output, multiple drains, and medications such as corticosteroids and statins
Manganese	Not well documented	Neuropsychiatric symptoms, Parkinson-like signs	Whole blood manganese, serum manganese Magnetic resonance imaging (MRI) can reveal deposition in the basal ganglia in toxicity	Cholestasis	N/A

N/A: not applicable.
